# Assessing the Potential of Rare Earth Elements in Bottom Ash from Coal Combustion in Poland

**DOI:** 10.3390/ma17174323

**Published:** 2024-08-31

**Authors:** Zdzisław Adamczyk, Joanna Komorek, Barbara Białecka, Jacek Nowak

**Affiliations:** 1 Faculty of Mining, Safety Engineering and Industrial Automation, Silesian University of Technology, ul. Akademicka 1, 44-100 Gliwice, Poland; joanna.komorek@polsl.pl; 2 Department of Environmental Monitoring, Central Mining Institute – National Research Institute, Plac Gwarków 1, 40-166 Katowice, Poland; bbialecka@gig.eu

**Keywords:** bottom ash, rare earth elements, critical raw materials

## Abstract

The aim of the research was to assess the potential of bottom ash from Polish coal-fired power plants as an alternative source of rare earth elements (REY). The potential of these ashes was compared with fly ash from the same coal combustion cycle. The phase and chemical composition, as well as REY, were determined using: X-ray diffraction and inductively coupled plasma mass spectrometry. The tested ashes were classified as *inert-low pozzolanic* and *inert-medium pozzolanic*, as well as *sialic* and *ferrosialic*, with enrichment in detrital material. The phase and chemical composition of bottom ash was similar to fly ash from the same fuel combustion cycle. The REY content in the ash was 199–286 ppm and was lower than the average for global deposits, and the threshold value was considered profitable for recovery from coal. Bottom ash’s importance as a potential source of REY will increase by recovering these metals from separated amorphous glass and mullite and grains rich in Al, Mg, K, and P. The industrial value of bottom ash as an alternative source of REY was similar to fly ash from the same fuel combustion cycle.

## 1. Introduction

Maintaining global industrial production at current levels is linked to a high energy demand, which is still mainly supplied by fossil fuels. This type of energy production is associated with atmospheric and other emissions into the environment. However, renewable energy has become a beacon of hope for our civilization and can provide an alternative to fossil fuels or a type of replacement, especially in the development of technologies such as photovoltaics, wind turbines, and electric vehicles. Rare earth elements (REEs) are needed for such technological developments, as well as for electronics, telecommunications, metallurgy, the chemical industry, medicine, defense, and others. It is common knowledge that these are at a high risk of shortages and market disruptions due to their rarity in the Earth’s crust [1,2,3,4,5,6,7,8,9]. In 1787, Ytterby, Sweden, pioneered the study of REEs, with the discovery of the new mineral ytterbite, a black rock, by Carl Axel Arrhenius. The first of the elements of this group, yttrium, was only found in this rock by the Finnish chemist Johan Gadolin in 1794. The period of the first industrial applications of REEs was between 1891 and 1930. In 1903, the Austrian chemist Carl Auer von Welsbach was the first to apply cerium to the ferrocerium alloy, which was used as an ignition source for lighters. This alloy is still produced today. The 1960s was the advent of a period of rapid development in the field of REE applications, driven by the discovery of their unique properties (magnetic, optical, and catalytic) [4,10,11,12,13,14].

The distinctive characteristics of these metals have facilitated the advancement of numerous fields, where electronic technology has been facilitated by the miniaturization of devices; the production of batteries has been enhanced by the incorporation of higher energy density, improved discharge characteristics, and simplified disposal; the utilization of catalysts has been expanded into the chemical, refining, and automotive industries; the glass industry has benefited from the incorporation of additional functions into glass; laser manufacturing; metallurgy industry (alloys with specific properties); ceramics; defense and satellite systems; alternative energy sources; and energy-saving technologies [15,16,17,18,19,20].

The convenience of modern and innovative technologies has increased the demand for REEs in world markets. Before the 1950s, the annual world production of REOs (REE oxides) was below 5000 Mg. However, in the 1960s, their use and demand began to increase, and their consumption increased by a factor of 5. Since the 1970s, the production and consumption of REEs have increased rapidly, reaching 350,000 Mg in 2023 [21,22,23]. Forecasts indicate that REE demand will continue to rise and peak its global production in 2041 by approx. 247,000 Mg. However, previous forecast scenarios indicated that the demand in 2035 will be 450,000 Mg [7]. The total REO reserves are estimated to be approx. 110 million Mg [23], which equates to a static depletion rate (reserves/current production) of 314 years. This approximation contrasts with earlier estimates of 870 years [24]. REE production is based on natural resources found in the Earth’s crust. These include various types of deposits, such as carbonatites and alkaline igneous rocks, skarn, pegmatites, laterites, ion adsorption clays, placer deposits, hydrothermal, and marine sediments. However, upon the extraction of these resources, their supply will inevitably decline. The reliability of the REE supply chain is therefore of paramount importance for economic development, however there is a growing concern about the stability of the market in this regard. Consequently, research is being conducted to identify alternative sources of REEs, with the potential extraction of these metals from power generation fly ash being a promising avenue of exploration. Fly ash has several important advantages over REE ores: firstly, it is readily available in large quantities as waste; secondly, it does not require special mining inputs; and thirdly, it is a powder material that is easy to process. However, the REE content of fly ash varies depending on the region, which stems the geological processes shaping coal deposits. These variations range from tens of ppm to 1% [9,25,26,27,28,29,30,31,32,33,34,35,36,37,38,39,40,41,42,43,44,45,46,47].

The potential of bottom ash as an alternative source of REEs has received less attention than that of fly ash. Several publications have reported REE contents in bottom ash, which range from 49 to 1203 ppm [27,48,49,50,51,52,53]. However, it should be noted that, although accounting for a much smaller proportion than fly ash during thermal coal combustion, bottom ash has an REE content comparable to fly ash.

The production of REEs from natural resources or alternative sources is a multi-step process with environmental risks. A growing body of research considers the recovery of REEs and the circular economy [12,17,18,20,54,55,56,57]. It can be concluded that both fly ash and bottom ash, of attractive concentrations, can provide an alternative source of REEs to natural resources. In this case, the entire process of mining ores and their preparation for the extraction of metals is circumvented, thereby reducing the environmental impacts associated with mining operations and ore processing to extract these metals.

REE include lanthanides (15 elements) along with scandium and yttrium. Yttrium is closely related to lanthanides (similar ionic radius and ionic charge), hence they are referred to as REY. In the geochemical division of REY, three groups are distinguished [32,58,59]: light—(LREYs: La, Ce, Pr, Nd, Pm, and Sm), medium—(MREYs: Eu, Gd, Tb, Dy, and Y), and heavy—(HREYs—Ho, Er, Tm, Yb, and Lu). The position of yttrium is between Dy and Ho. A market division has also been adopted based on the forecasted relationships between the demand and supply of the following groups of elements [59]: critical (Nd, Eu, Tb, Dy, Y, and Er), uncritical (La, Pr, Sm, and Gd), and excessive (Ce, Ho, Tm, Yb, and Lu).

REY contents in fly ash or bottom ash are usually reported, but the results of these wastes originating from the same fuel combustion cycle are rarely presented. The aim of our study was to show the distribution of REY in bottom ash samples and compare it to the distribution in fly ash for samples of both wastes collected during the same combustion cycle in several Polish coal-fired power plants. Additionally, we assessed the industrial potential of bottom ash and indicated the justification for increasing its importance as an alternative source of REY. Furthermore, a comparison study was conducted on the potential of bottom ash with the potential of fly ash from the same coal combustion cycle.

## 2. Materials and Methods

Eight bottom ash samples were collected for the study from four Polish power plants equipped with conventional (pulverized coal) boilers (Table 1). The ashes were generated from the combustion of hard coal. The samples were labeled Z1–Z8.

Two samples were collected at each power station (from two different power units) over a period of one month at a rate of approx. 10 kg per day. Subsequently, the samples were reduced in size and transported to the laboratory. The material was then subjected to grinding in a zircon mill and sieving through a sieve with a mesh of less than 0.10 mm.

The phase composition, main chemical constituents, and REY were determined in the material prepared for testing.

The phase composition was determined by X-ray diffraction using an Empyrean diffractometer from PANalytical (Malvern, UK). The measurements were conducted under the following conditions: The X-ray diffraction measurements were conducted using a Cu lamp with an angular range from 5° to 70°, a step size of 0.02°, and an exposure time of 2 s. The proportions of the different phases were determined by the Rietveld method using HighScore Plus software (version 4.9).

The main chemical components, including SiO_2_, TiO_2_, Al_2_O_3_, Fe_2_O_3_, Mn_3_O_4_, MgO, CaO, Na_2_O, K_2_O, P_2_O_5_, and SO_3_, as well as the content of REY, were determined using inductively coupled plasma mass spectrometry (ICP-MS). The samples were mixed with a flux of lithium metaborate and lithium tetraborate and fused in an induction furnace. Molten melt was immediately poured into a solution of 5% nitric acid containing an internal standard and mixed continuously until completely dissolved. The prepared solutions were analyzed. The measurements were obtained using a Perkin Elmer SCIEX ELAN 6000 ICP-MS spectrometer at Activation Laboratories Ltd. in Ancaster, ON, Canada.

The prospective coefficient (C_outl_) was calculated according to the following formula [32]:

(1) $$C_{outl}=\frac{Nd+Eu+Tb+Dy+Er+Y}{Ce+Ho+Tm+Yb+Lu}$$

The bottom ashes under investigation were evaluated as a potential alternative source of REY, with the industrial value of the material determined based on the dependence of the percentage of critical elements on C_outl_.

The REY contents of the samples were normalized to their contribution to the upper continental crust (UCC) in order to determine the degree of enrichment of the studied samples relative to the UCC. There are three types of enrichment, which depend on the distribution of REY content according to subgroups [32,58,59]: Type L—enrichment in LREY (La + Ce + Pr + Nd + Pm + Sm); Type M—enrichment in MREY (Eu + Gd + Tb + Dy + Y); and Type H—enrichment in HREY (Ho + Er + Tm + Yb + Lu), which depends on the value of ratios: LaN/LuN > 1 (type L); LaN/SmN < 1 and GdN/LuN > 1 (type M); and LaN/LuN < 1 (type H).

## 3. Results

### 3.1. Phase Composition

Despite the samples originating from different power plants, the phase composition of the bottom ashes exhibited a remarkably consistent inventory of components. These included silicates (quartz, mullite, and anorthite), iron oxides (hematite, magnetite, and maghemite), Fe-containing spinels, calcite, and a glassy phase. The study demonstrated that the predominant component of the analyzed ashes was the glass phase, with an average proportion of 62.6%. The lowest contents were observed in ashes from the Łagisza power station (52.0–59.1%), while the highest were found in ashes from the Jaworzno III power station (Table 2). The second most prevalent component was mullite, with an average content of 20.1% in the analyzed ashes. The lowest contents were observed in samples from the Siersza power station (11.9–17.2%), while the highest was from the Łagisza power station (23.9–27.7%). Another component in the analyzed ash in higher amounts was quartz, with an average content of approx. 10%. The lowest amounts were present in ash from the Łaziska power station (4.3–5.7%) and the highest from the Siersza power station (11.2–15.7%). The quantities of the other phase components were considerably lower, with no values exceeding 5%. The three components (glassy phase, mullite, and quartz) collectively account for more than 90% of the samples.

**Figure 1:**
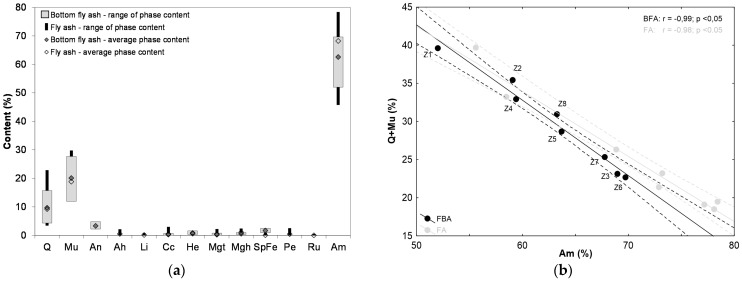
Differentiation of the contents of: (**a**) individual phases; (**b**) dependence of the glassy phase (Am) on the sum of quartz and mullite (Q + Mu) in the tested bottom ash (BFA) and fly ash (FA) (*r*—correlation coefficient value; *p*—confidence interval).

A comparison of the amounts of the individual phase components in the bottom ashes under investigation with that of components in previously studied fly ash samples from the same power plants and collected during the same fuel combustion cycle [42] indicated that the proportions of the dominant phases, namely glass phase, mullite and quartz, in the fly ashes were significantly higher than in the bottom ashes (Figure 1a). A clear relationship between the proportion of the glass phase (Am) and the sum of the amounts of quartz and mullite (Q + Mu) was apparent in both the bottom ash and fly ash (r = 0.99, *p* < 0.05; r = 0.98, *p* < 0.05, respectively). This highlighted that the observed increase in the proportion of Q + Mu was associated with a reduced Am amount (Figure 1b).

**Figure 2:**
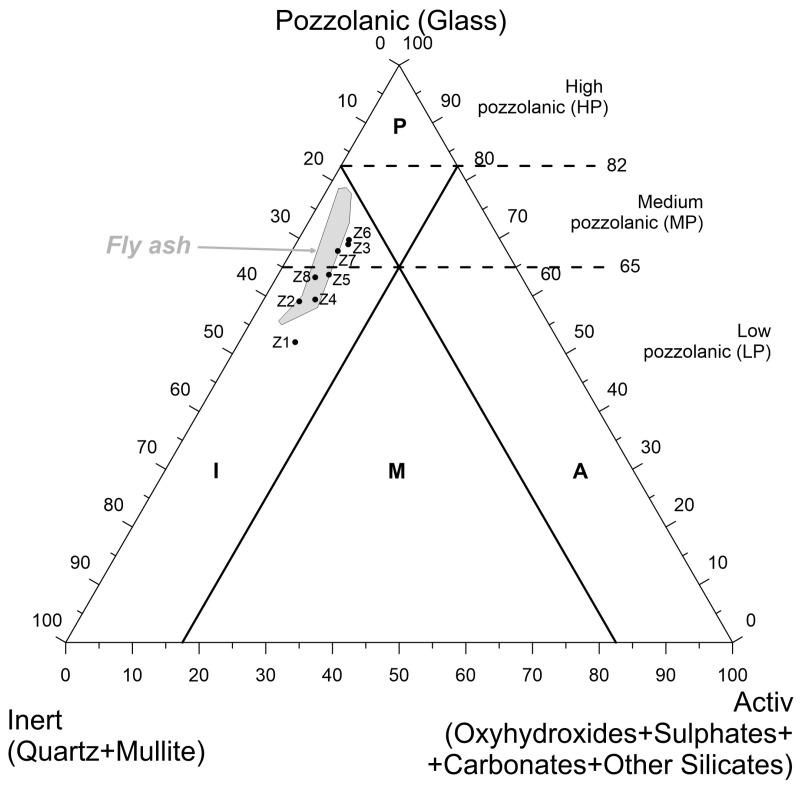
Projection points of the tested bottom ashes in Vassilev’s phase classification of fly ash [60] against the background of fly ash from the same fuel combustion cycle [42].

In Vassilev’s classification [60], which considers the quantities of components such as *activ* (oxyhydrooxides + sulfates + carbonates + other silicates), *puzzolanic* (glass), and *inert* (quartz + mullite), the examined ashes of both samples from the Lagisza power plant are classified as *inert-low pozzolanic* (I-LP). The remaining ash samples exhibited ambiguous positions, with the majority falling within the *inert-low pozzolanic* (I-LP) or *inert-medium pozzolanic* (I-MP) fields (Figure 2). Therefore, the fuel fed to the power boilers exhibited some degree of variability in quality, although theoretically, such a variation should not have occurred. It was observed that the projection points of bottom ash in Vassilev’s diagram were arranged along the *Pozzolanic*-*Insert* line, which was almost identical to the projection field for fly ash that came from the same power plants and was collected in the same fuel combustion cycle [42].

### 3.2. Chemical Composition

The chemical composition of the bottom ashes was analyzed, and the results reveal that the main constituents are SiO_2_, Al_2_O_3_, and loss of ignition (LOI), with average contents of 47.81%, 22.82%, and 9.05% mass, respectively (Table 3). It was notable that the samples from the Łaziska power plant exhibited a slightly higher content of Al_2_O_3_ compared to the other samples. This was evidenced by the lowest value of the SiO_2_/Al_2_O_3_ ratio, which was 1.84–1.88. The aforementioned ratio was observed to be above 2.00 in the remaining samples, with values as high as 2.40–2.50 being observed in the Siersza power plant samples. The SiO_2_ and Al_2_O_3_ contents exhibited minimal variation, with coefficients of variation of 3.59% and 10.39%, respectively. A markedly greater degree of variation was observed for LOI, with a range of 0.79–16.55% mass and a coefficient of variation exceeding 59%. The energy waste from pulverized coal-fired boilers is primarily attributable to the presence of unburned fuel. The lowest LOI contents in the investigated ash, as shown in the Jaworzno III and Łaziska power plants (0.79–5.35% and 3.19–7.66% masses, respectively), were attributed to a more efficient combustion process or superior fuel quality in comparison to the other power plants.

Fe_2_O_3_ was of particular interest among the other chemical components, which exhibited an average content below 10% mass. However, only the samples from the Jaworzno III power plant had amounts above this value, ranging from 10.06% to 15.35% mass.

The average contents of MgO, CaO, Na_2_O, and K_2_O were a few mass percent each (2.40%, 3.29%, 1.18%, and 2.35% mass, respectively). However, none of the samples exceeded a 4% mass. The calculated CaO/MgO ratios ranged from 1.16 to 1.71 (mean 1.37), while the K_2_O/Na_2_O ratios were in the range of 1.17–4.22 (mean value of 2.22).

TiO_2_ content was approx. 1% mass, while that of Mn_3_O_4_, P_2_O_5_, and SO_3_ did not exceed 0.50% mass, with averages of 0.10%, 0.20%, and 0.25% mass, respectively.

The results of the chemical composition tests allowed for the classification of the tested bottom ash according to ASTM C618 (2015) into class F [61], as evidenced by the following criteria: (i) the sum of SiO_2_, Al_2_O_3_, and Fe_2_O_3_ oxides was above 70% mass, and (ii) the CaO content was below 20% mass. The obtained results reveal that, according to the Polish classification, they belong to siliceous ash (k) as they meet the following criteria: (i) SiO_2_ > 40%; (ii) Al_2_O_3_ < 30%; (iii) CaO <10%; and (iv) SO_3_ < 4%.

**Figure 3:**
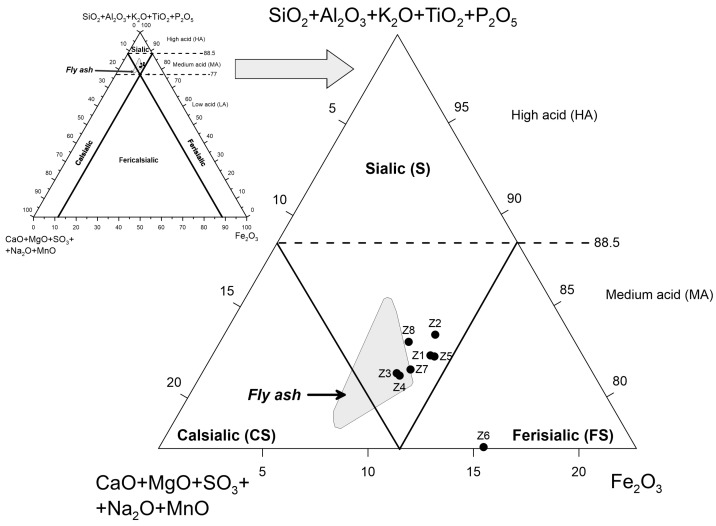
Projection points of the tested bottom ashes in Vassilev’s chemical classification of fly ash [60] against the background of fly ash from the same fuel combustion cycle [42].

The projection of the points of the studied ashes in the Vassilev classification system based on the ratio of *Fe*_2_*O*_3_*-SiO*_2_ *+ Al*_2_*O*_3_ *+ K_2_O + TiO*_2_ *+ P*_2_*O*_5_*-CaO + MgO + SO*_3_ *+ Na*_2_*O + MnO* [60] revealed that they generally belonged to the *sialic*-medium acid (S-MA) type (Figure 3). The exception was a sample from the Jaworzno III power plant (Z6), which was classified as a *ferrisialic*-medium acidic (FS-MA) type. This sample exhibited the highest Fe_2_O_3_ content (15.35% mass). The positions of the points on the diagram were a logical consequence of the significant amounts of identified phase components, namely quartz and mullite, which belong to silicates, aluminosilicates, and glass, and are usually dominated by SiO_2_ and Al_2_O_3_.

The projection points of the bottom ash in the Vassilev’s chemical ash classification diagram (Figure 3) were situated within or close to the projection field for fly ash from the same power. They were obtained during the same fuel combustion cycle [42]. Points outside the fly ash field demonstrated a shift toward the top of the *SiO*_2_ *+ Al*_2_*O*_3_ *+ K*_2_*O + TiO*_2_ *+ P*_2_*O*_5_ component, indicating a higher proportion of the sum of these components and a lower proportion of the sum of *CaO + MgO + SO*_3_ *+ Na*_2_*O + MnO* components compared to points in the field. The sole exception was sample Z6, located outside the fly ash characteristic field. This sample exhibited a shift toward the top of *Fe*_2_*O*_3_, thereby indicating an increase in the contribution of this component.

It was found that some of the chemical components exhibited a high positive or negative correlation with one another, with the absolute value of *r* exceeding 0.70 and *p* being less than 0.05. These included SiO_2_-LOI (−0.81), and TiO_2_-Al_2_O_3_ and LOI (0.91 and −0.80, respectively); Al_2_O_3_-K_2_O, P_2_O_5_, and LOI (0.76, 0.88, and −0.74, respectively); Fe_2_O_3_-SO_3_ and LOI (0.76 and −0.73, respectively); Mn_3_O_4_-MgO and K_2_O (0.85 and 0.78, respectively); MgO-CaO and K_2_O (0.71 and 0.78, respectively); Na_2_O-P_2_O_5_ and SO_3_ (−0.78 and −0.71, respectively); K_2_O-P_2_O_5_ (0.82); and SO_3_-LOI (−0.72). Correlations of a similar nature (r > |0.70|, *p* < 0.05) were observed for the following variables: SiO_2_/Al_2_O_3_-TiO_2_, K_2_O, and P_2_O_5_ (−0.79, −0.71, and −0.96, respectively); CaO/MgO-Fe_2_O_3_ (0.77); and K_2_O/Na_2_O-P_2_O_5_ (0.87).

The calculated detrital/authigenic index (DAI)—(SiO_2_ + Al_2_O_3_ + K_2_O + Na_2_O + TiO_2_)/(Fe_2_O_3_ + CaO + MgO + SO_3_ + P_2_O_5_ + MnO) ranged from 3.51 to 5.38 (mean 4.80). These values unambiguously demonstrated that the ashes under investigation were formed through the combustion of coal containing detrital mineral matter [60]. The DAI index exhibited a high negative correlation with Fe_2_O_3_ (r = −0.90, *p* < 0.05) and SO_3_ (r = −0.72, *p* < 0.05), as well as with (MgO + CaO)/(K_2_O + Na_2_O) ratios (r = −0.87, *p* < 0.05) and CaO/MgO (r = −0.84, *p* < 0.05).

### 3.3. Rare Earth Elements

The concentrations of individual REY in the studied bottom ash samples exhibited slight variability (Table 4), similar to the total REY, for which the coefficient of variation was V = 12.31%. Indeed, the contents of REY ranged from 199.61 to 286.01 ppm, with the highest concentrations observed in bottom ash from the Łaziska power plant. The mean REY of 234.72 ppm was below the mean for world deposits [32,62,63]. The converted REY contents to REO oxides, for which the average was 277.72 ppm, are also lower than the average for coal ash of world deposits, and below the value of 1000 ppm, which is considered to be the limit for cost-effective recovery from coal.

It is worth noting that the sample contained a relatively high concentration of Ce (mean = 78.81 ppm), as well as elevated levels of Nd, Y, and La in comparison to other elements (mean = 37.63, 37.46, and 34.79 ppm, respectively). Consequently, the distribution of REY in coal and coal ash could be described in terms of three subgroups of elements [32,58,59], with the LREY subgroup being the dominant one, as evidenced by the mean value of 168.75 ppm. This value accounted for more than 71% of the total REY. The mean shares of the remaining subgroups were MREY (23.68%) and HREY (4.47%).

Additionally, the results indicate that the critical element contents range from 75.30 to 109.70 ppm in the samples (mean value of 89.35 ppm), which are comparable to the excess element contents at 72.71–104.81 ppm and s = 85.31 ppm. Consequently, the mean contributions of these two elemental groups to the total REY content were 38.08% for the critical and 36.33% for the excessive elements, with low variability (2.86 and 2.05%, respectively).

Non-critical elements exhibited slightly lower contents than critical and excessive elements, with a range of 51.60–71.50 ppm (mean value of 60.06 ppm). This resulted in their lower contribution to the total REY content, with a mean value of 25.60 ppm and low variability (V = 2.32%).

**Figure 4:**
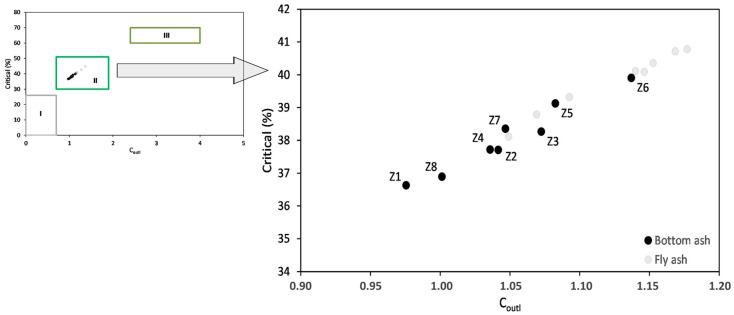
The relationship between the percentage of critical elements in the tested bottom ashes and the prospective C_outl_ coefficient compared to the classification of coal ashes enriched in REEs [32] against the background of fly ash from the same fuel combustion cycle [42]. REY source: I—non-prospective; II—prospective; III—a highly prospective REY source.

The prospective C_outl_ factor, calculated based on the determined contributions of critical and excessive elements, exhibited a narrow range of values, ranging from 0.98 to 1.14, with low variability (V = 4.74%). In terms of their potential industrial value, the bottom ashes were assessed based on their individual REY composition. As an alternative source of these elements, the percentage of critical elements was found to depend on C_outl_. Therefore, the bottom ashes were placed in cluster II, which was defined as a promising raw material for REY (Figure 4). The same assessment of the potential industrial value was established for fly ash from the same power plants and taken during the same fuel combustion cycle [42].

**Figure 5:**
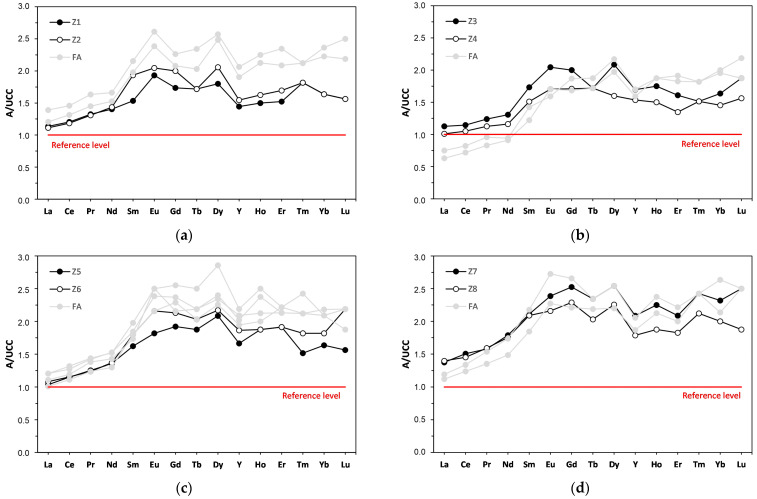
Distribution of REY content in the tested bottom ash against the background of fly ash (FA) from the same fuel combustion cycle according to power plants: (**a**) Łagisza; (**b**) Siersza; (**c**) Jaworzno III; (**d**) Łaziska. The proportion of REY was normalized to their content in the upper continental crust (UCC) [64].

The calculated La_N_/Lu_N_, La_N_/Sm_N_, and Gd_N_/Lu_N_ ratios indicated that all the studied bottom ash samples exhibited an H-type REY distribution relative to the UCC, thereby demonstrating enrichment in the HREY subgroup of heavy elements. All standardization curves were above the reference level, and the contents of individual elements were typically twice as high as those of the UCC [64] (Figure 5). The normalization curves showed similar enrichment and characteristics of the analyzed fly ash from the same coal combustion cycle [42].

## 4. Discussion

It is important in the field of REY recovery technologies to ascertain which minerals act as carriers of these elements, or to determine the relationship between the chemical compound and these elements [9,65]. In order to highlight these relationships, a correlation analysis was performed between the following variables: the proportion of identified phases with chemical constituents; REY elements and their subgroups (LREY, MREY, and HREY), as well as critical, non-critical, and excess with phase compositions; and REY elements and their subgroups (LREY, MREY, and HREY), as well as critical, non-critical, and excess with chemical constituents.

### 4.1. Correlation between Phase Components and Chemical Components

Some of the identified phases exhibited a significantly high positive or negative correlation with the chemical components, with an absolute value of r > 0.70, *p* < 0.05. The following correlations were observed: quartz-TiO_2_ and Al_2_O_3_ (0.80 and −0.92, respectively), anorthite-Na_2_O and P_2_O_5_ (0.83 and −0.77, respectively), amorphous substance-SiO_2_ (0.73), quartz-K_2_O/Na_2_O (−0.76), and mullite-CaO/MgO (−0.80).

### 4.2. The Relationship between REY and Phase Components

**Figure 6:**
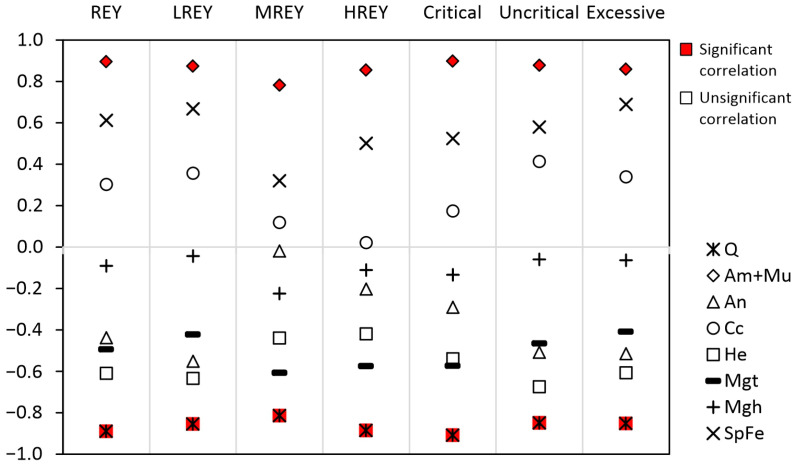
Values of correlation coefficients for the relationships between the contents of REY, LREY, MREY, HREY, and critical, non-critical, and excessive elements, and the contents of some phases in the tested bottom ash. Definitions: Am, glass; An, anorthite; Cc, calcite; He, hematite; Mgh, maghemite; Mgt, magnetite; Mu, mullite; Q, quartz; SpFe, Fe spinel.

**Figure 7:**
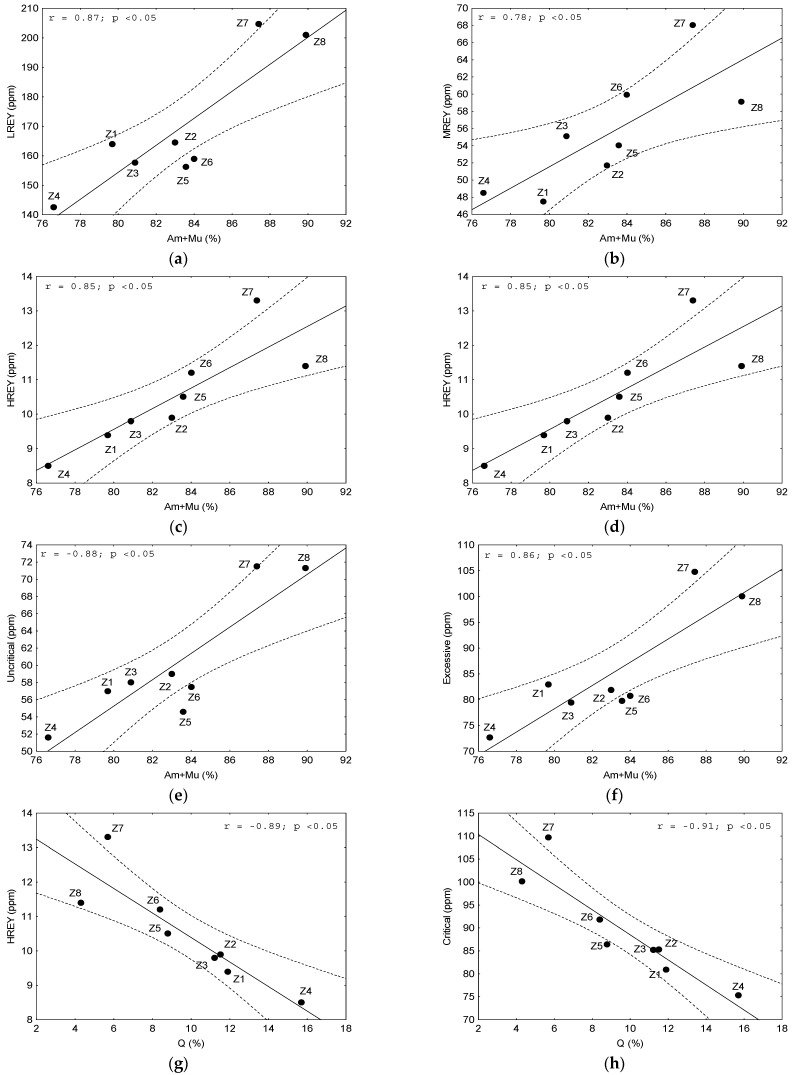
Dependence on the contents of: (**a**–**f**) LREY, MREY, HREY, and critical, non-critical, and excessive elements on the sum of glass and mullite (Am+Mu); (**g**,**h**) HREY and critical elements on quartz (Q) in the tested bottom ash. Explanations: *r*—correlation coefficient value; *p*—confidence interval.

The correlation coefficients between REY and its subgroups, together with the critical, non-critical, and excess elements with the phase components, indicated (Figure 6) a strong and significant positive correlation from the sum of mullite and amorphous glass (r = 0.78–0.90, *p* < 0.05) (Figure 7a–f). The combined treatment of these two components was justified, as mullite is typically dispersed within the grains of the amorphous glass and rarely forms independent grains. Furthermore, REY elements are typically dispersed in ashes within the vitreous phase [35,37,65,66]. Therefore, it was necessary to consider the separation of these phase components to extract REY components.

The second phase demonstrated a robust and statistically significant inverse correlation with all the aforementioned REY element groups with the strongest correlation being observed for quartz (r = −0.81 to −0.91, *p* < 0.05) (Figure 7g,h). This relationship between REY and quartz was expected as REY is unable to be incorporated into the quartz structure due to its crystallochemical properties. Furthermore, the preferred form of their presence in quartz is liquid inclusions [67,68], which are excluded for ash. Consequently, the removal of quartz from ash may result the enrichment of REY.

It was noted that other phase components did not exhibit a strong correlation with REY element groups. In certain cases, the absolute values of this coefficient were above 0.60, but they were not statistically significant (e.g., Fe spinels with REY, LREY, and excess elements; hematite with REY, LREY, non-critical, and excess elements; and magnetite with MREY) (Figure 6).

### 4.3. The Relationship between REY and Chemical Components

**Figure 8:**
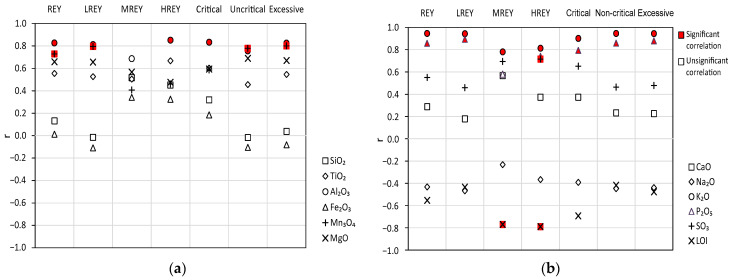
Values of correlation coefficients (*r*) for the relationships between the contents of REY, LREY, MREY, HREY, and critical, non-critical, and excessive elements, and the contents of (**a**) SiO_2_, TiO_2_, Al_2_O_3_, Fe_2_O_3_, Mn_3_O_4_, and MgO; (**b**) CaO, Na_2_O, K_2_O, P_2_O_5_, SO_3_, and LOI in the tested bottom ash.

**Figure 9:**
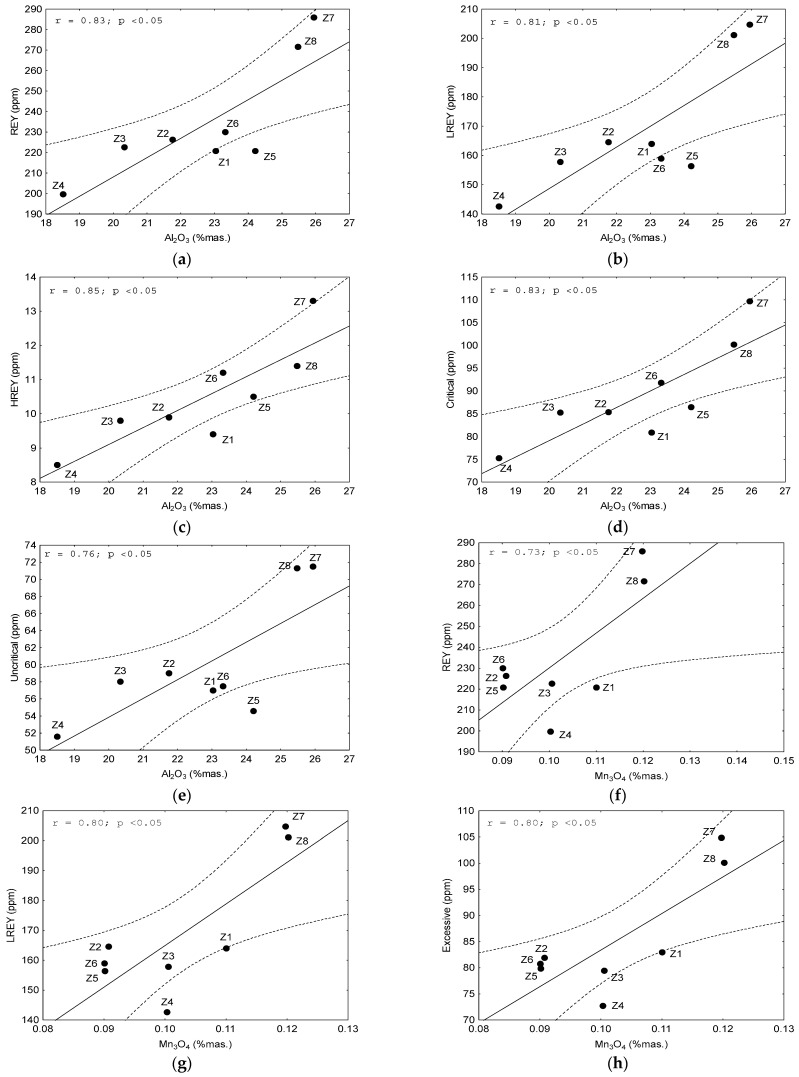
Dependence on the content of: (**a**–**e**) REY, LREY, MREY, and critical and non-critical elements on the Al_2_O_3_; (**f**–**h**) REY, LREY, and excessive elements on Mn_3_O_4_ in the tested bottom ash. Explanations: *r*—correlation coefficient value; *p*—confidence interval.

**Figure 10:**
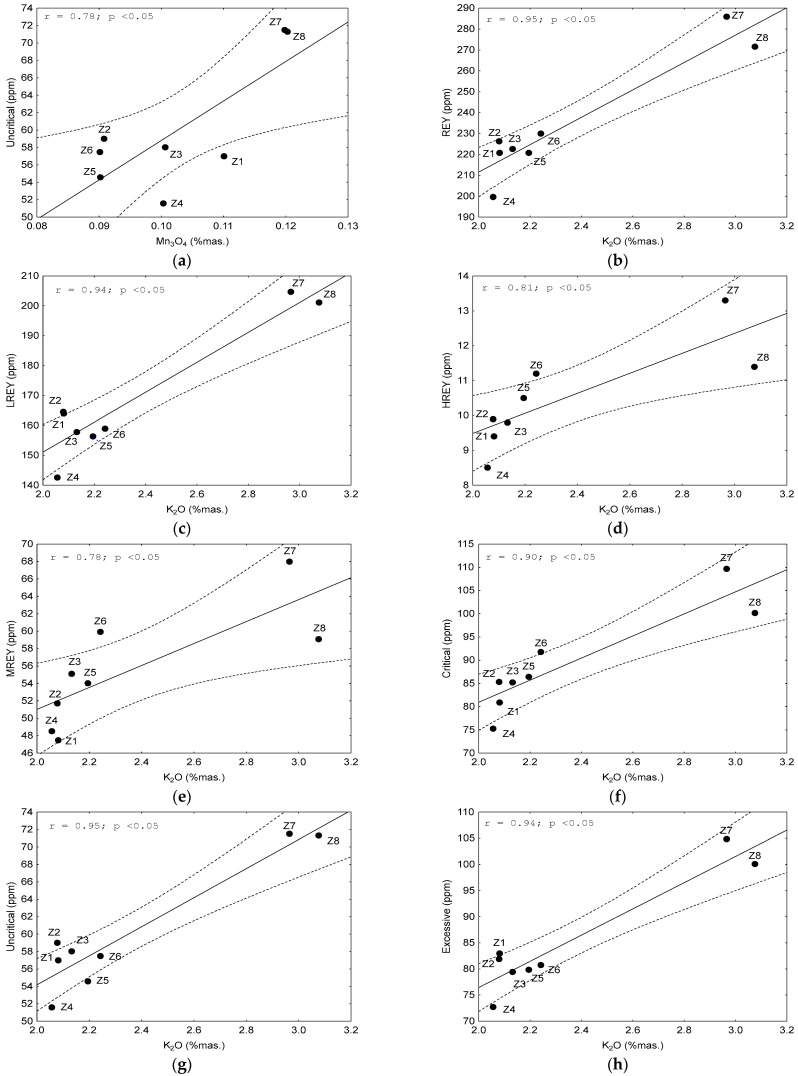
Dependence on the content of: (**a**) non-critical elements on the Mn_2_O_3_; (**b**–**h**) REY, LREY, critical, non-critical, and excessive elements on K_2_O in the tested bottom ash. Explanations: *r*—correlation coefficient value; *p*—confidence interval.

**Figure 11:**
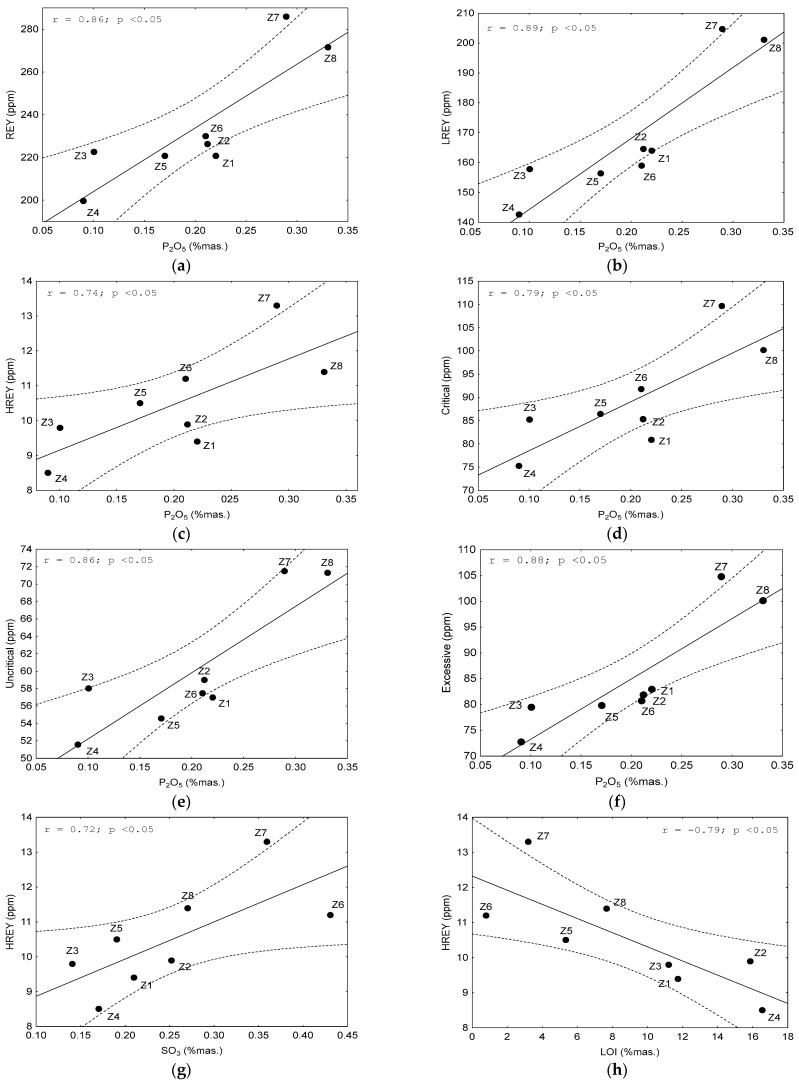
Dependence on the content of: (**a**–**f**) REY, LREY, HREY, critical, non-critical, and excessive elements on P_2_O_5_; (**g**) HREY on SO_3_; (**h**) HREY on the LOI in the tested bottom ash. Explanations: *r*—correlation coefficient value; *p*—confidence interval.

REY elements and their subgroups (LREY, MREY, and HREY), as well as critical, non-critical, and excessive elements, demonstrated a strong and significant correlation with some chemical components with an absolute value of the correlation coefficient r > 0.70 at *p* < 0.05. The following elements were found to be significantly correlated (i) with REY and LREY: Al_2_O_3_, Mn_3_O_4_, K_2_O, and P_2_O_5_, (ii) with MREY: K_2_O and LOI; (iii) with HREY: Al_2_O_3_, K_2_O, P_2_O_5_, SO_3_, and LOI; (iv) with critical elements: Al_2_O_3_, K_2_O, and P_2_O_5_; and (v) with non-critical and excessive elements: Al_2_O_3_, Mn_3_O_4_, K_2_O, and P_2_O_5_ (Figure 8). We observed from the above data that the individual REY subgroups, as well as the critical, non-critical, and excessive elements, tended to correlate with the recurring chemical components. This mainly includes Al_2_O_3_ (Figure 9a–e), Mn_3_O_4_ (Figure 9f–h, 10a), K_2_O (Figure 10b–h), and P_2_O_5_ (Figure 11a–f), and occasionally with SO_3_ (Figure 11g) and LOI (Figure 11h).

**Figure 12:**
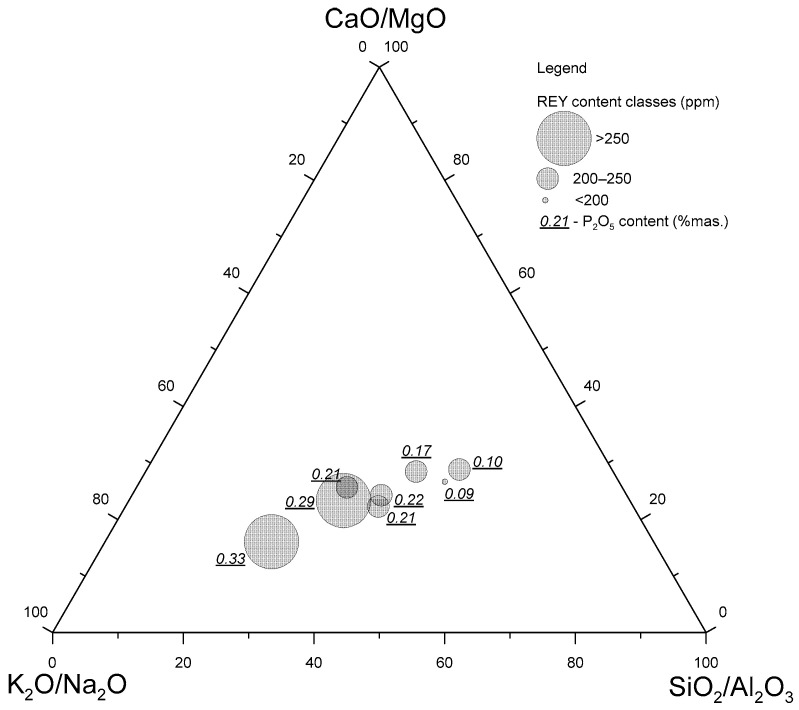
Diagram of the *SiO_2_/Al_2_O_3_-CaO/MgO-K_2_O/Na_2_O* system, with projection points indicating the range of REY and P_2_O_5_ contents in the bottom ash samples tested.

A strong and significant correlation was observed between the ratios of some of the components and the individual REY element groups. This correlation was negative for SiO_2_/Al_2_O_3_ with all REY groups, and positive for K_2_O/Na_2_O with REY, LREY, and non-critical elements. An *SiO*_2_*/Al*_2_*O*_3_*-CaO/MgO-K*_2_*O/Na*_2_*O* diagram was constructed, presenting the chemical compositions and correlations between the individual components, with projection points indicating the range of the REY content and P_2_O_5_ content in the samples (Figure 12). We observed a concentration of projection points with higher REY and P_2_O_5_ contents toward *K*_2_*O/Na*_2_*O* and with lower contents of these components toward the *SiO*_2_*/Al*_2_*O*_3_*-CaO/MgO* line. A higher proportion of potassium with a lower proportion of sodium and, at the same time, a higher proportion of aluminum with less silicon and higher magnesium with less calcium is indicative of an increase in phosphorus and REY elements. It can be concluded that the higher the concentrations of aluminum, magnesium, potassium, and phosphorus, the higher the proportion of REY in the bottom ash.

The associations of REY elements and their subgroups, together with critical, non-critical, and excess elements with the phase and chemical compositions in the bottom ashes studied only for Al_2_O_3_ and P_2_O_5_ were found to be similar to those observed in fly ashes from the same fuel combustion cycle [42]. Nevertheless, the quantitative characterization of the different elemental groups showed that the bottom ash and fly ash were comparable. However, small differences in their phase and chemical composition indicated some correlations did not repeat (e.g., TiO_2_, MgO, CaO, and DAI) or adopt the opposite sign in bottom ash (Mn_3_O_4_, sometimes K_2_O and Na_2_O). This may indicate the occurrence of a specific geochemical differentiation on a small scale in the formation conditions of these two energy wastes, which is difficult to explain unambiguously.

## 5. Conclusions

The studies conducted on bottom ash from the combustion of hard coal in Polish power plants revealed that the dominant phases were mullite, quartz, and amorphous glass. These phases, in conjunction with the other phases, permitted the classification of the ash as *inert-low pozzolanic* and *inert-medium pozzolanic* types, which made them comparable fly ash from the same fuel combustion cycle.

The principal chemical constituents of the bottom ashes under investigation were SiO_2_ and Al_2_O_3_. Considering the remaining constituents permitted the classification of the ashes into the *sialic* and *ferrosialic* types, with enrichment in detrital material. Hence, the ashes were comparable to the fly ashes from the same fuel combustion cycle.

The REY content in the bottom ash was in the range of 199–286 ppm, which was lower than the average for global deposits. Furthermore, the REY content converted into oxides was lower than the average for coal ash from global deposits and lower than the limit considered viable for recovery from coal (1000 ppm). A distribution of H-type REY was observed relative to the UCC, i.e., with enrichment in the heavy element subgroup HREY. Similar characteristics were noted for fly ash from the same fuel combustion cycle.

The potential industrial value of the studied bottom ashes was assessed based on the calculated values of the prospective C_outl_ factor and the individual REY composition. This indicates that the bottom ashes could be considered as an alternative source of REY and defined as REY promising raw materials.

In order to enhance the significance of bottom ash as a potential source of REY and to increase the concentration of these elements, the recovery of these metals should be carried out from separated amorphous glass together with mullite and grains rich in aluminum, magnesium, potassium, and phosphorus.

The results of the conducted studies indicate that the assessment of the potential industrial value of the studied bottom ash from Polish coal-fired power plants as an alternative REY source (a promising raw material) is in many respects similar to the potential of fly ash from the same fuel combustion cycle.

## Figures and Tables

**Table 1:** The selected bottom ash samples for testing.

Power Plant	Sample
Łagisza	Z1
	Z2
Siersza	Z3
	Z4
Jaworzno III	Z5
	Z6
Łaziska	Z7
	Z8

**Table 2:** The phase components content in the tested bottom ash samples (in % mass).

Power Plant	Sample	Q	Mu	An	Cc	He	Mgt	Mgh	SpFe	Am	Total
Łagisza	Z1	11.9	27.7	2.5	0.5	1.5	0.8	1.0	2.1	52.0	100.0
	Z2	11.5	23.9	2.6	0.4	0.4		0.6	1.5	59.1	100.0
Siersza	Z3	11.2	11.9	4.8	0.5	0.6		0.5	1.5	69.0	100.0
	Z4	15.7	17.2	3.7	0.7	1.1	0.7	0.3	1.2	59.4	100.0
Jaworzno III	Z5	8.8	19.9	4.2		1.0		0.3	2.1	63.7	100.0
	Z6	8.4	14.3	3.6	0.4	1.6	0.3	0.8	0.9	69.7	100.0
Łaziska	Z7	5.7	19.6	3.1	0.6	0.3		0.4	2.5	67.8	100.0
	Z8	4.3	26.6	2.2	0.7	0.3	0.1	0.5	2.0	63.3	100.0
Min.		4.3	11.9	2.2	0.0	0.3		0.3	0.9	52.0	
Max.		15.7	27.7	4.8	0.7	1.6	0.8	1.0	2.5	69.7	
S		9.7	20.1	3.3	0.5	0.9	0.2	0.6	1.7	62.6	
SD		3.4	5.3	0.8	0.2	0.5	0.3	0.2	0.5	5.8	
V		35.4	26.3	25.2	44.4	57.9	131.4	41.6	29.1	9.2	

Am, glass; An, anorthite; Cc, calcite; He, hematite; Mgh, maghemite; Mgt, magnetite; Mu, mullite; Q, quartz; SpFe, Fe spinel; Min., minimum; Max., maximum; S, average; SD, standard deviation; V, coefficient of variation.

**Table 3:** Content of main chemical components in the tested bottom ash (in % mass).

Power Plant	Sample	SiO_2_	TiO_2_	Al_2_O_3_	Fe_2_O_3_	Mn_3_O_4_	MgO	CaO	Na_2_O	K_2_O	P_2_O_5_	SO_3_	LOI	DAI	SiO_2_/ Al_2_O_3_	(MgO + CaO)/ (K_2_O + Na_2_O)	CaO/ MgO	K_2_O/ Na_2_O
Łagisza	Z1	46.37	0.91	23.04	9.19	0.11	2.23	2.86	1.05	2.08	0.22	0.21	11.73	4.96	2.01	1.63	1.28	1.98
	Z2	45.31	0.82	21.75	8.47	0.09	1.89	2.27	0.99	2.08	0.21	0.25	15.87	5.38	2.08	1.36	1.20	2.10
Siersza	Z3	48.80	0.81	20.34	8.26	0.10	2.57	3.72	1.82	2.13	0.10	0.14	11.21	4.96	2.40	1.59	1.45	1.17
	Z4	46.34	0.73	18.51	7.93	0.10	2.50	3.58	1.44	2.06	0.09	0.17	16.55	4.81	2.50	1.74	1.43	1.43
Jaworzno	Z5	50.23	1.01	24.21	10.06	0.09	2.08	2.97	1.45	2.19	0.17	0.19	5.35	5.08	2.07	1.39	1.43	1.51
	Z6	49.93	0.93	23.33	15.32	0.09	2.19	3.74	0.80	2.24	0.21	0.43	0.79	3.51	2.14	1.95	1.71	2.80
Łaziska	Z7	48.76	0.95	25.94	9.54	0.12	2.87	3.84	1.18	2.96	0.29	0.36	3.19	4.69	1.88	1.62	1.34	2.51
	Z8	46.81	0.96	25.48	8.32	0.12	2.89	3.35	0.73	3.08	0.33	0.27	7.66	5.04	1.84	1.64	1.16	4.22
Min.		45.31	0.73	18.51	7.93	0.09	1.89	2.27	0.73	2.06	0.09	0.14	0.79	3.51	1.84	1.36	1.16	1.17
Max		50.21	1.01	25.94	15.32	0.12	2.89	3.84	1.82	3.08	0.33	0.43	16.55	5.38	2.50	1.95	1.71	4.22
S		47.81	0.89	22.82	9.64	0.10	2.40	3.29	1.18	2.35	0.20	0.25	9.05	4.80	2.12	1.61	1.37	2.22
SD		1.71	0.09	2.37	2.25	0.01	0.34	0.51	0.35	0.39	0.08	0.09	5.39	0.56	0.23	0.19	0.17	0.98
V		3.59	9.84	10.39	23.37	11.51	14.23	15.62	29.16	16.59	38.35	36.51	59.63	11.66	10.98	11.67	12.57	44.22

LOI, loss of ignition; DAI, detrital/authigenic index; Min., minimum; Max., maximum; S, average; SD, standard deviation; V, coefficient of variation.

**Table 4:** The REY content in examined fly ash samples and basic statistical parameters.

PP	S	Y	La	Ce	Pr	Nd	Sm	Eu	Gd	Tb	Dy	Ho	Er	Tm	Yb	Lu	Type REE
		[ppm]															
Łaziska	Z1	31.80	34.10	77.04	9.40	36.50	6.90	1.70	6.60	1.10	6.30	1.20	3.50	0.60	3.60	0.50	H
	Z2	34.00	33.40	75.90	9.30	37.30	8.70	1.80	7.60	1.10	7.20	1.30	3.90	0.60	3.60	0.50	H
Siersza	Z3	37.30	33.80	73.35	8.80	34.00	7.80	1.80	7.60	1.10	7.30	1.40	3.70	0.50	3.60	0.60	H
	Z4	33.80	30.30	67.31	8.00	30.20	6.80	1.50	6.50	1.10	5.60	1.20	3.10	0.50	3.20	0.50	H
Jaworzno	Z5	36.60	31.10	73.72	8.90	35.30	7.30	1.60	7.30	1.20	7.30	1.50	4.40	0.50	3.60	0.50	H
	Z6	41.00	32.50	73.95	8.80	35.60	8.10	1.90	8.10	1.30	7.60	1.50	4.40	0.60	4.00	0.70	H
Łaziska	Z7	45.90	41.20	96.31	11.20	46.50	9.50	2.10	9.60	1.50	8.90	1.80	4.80	0.80	5.10	0.80	H
	Z8	39.30	41.90	92.89	11.30	45.60	9.40	1.90	8.70	1.30	7.90	1.50	4.20	0.70	4.40	0.60	H
Min.		31.80	30.30	67.31	8.00	30.20	6.80	1.50	6.50	1.10	5.60	1.20	3.10	0.50	3.20	0.50	
Max		45.90	41.90	96.31	11.30	46.50	9.50	2.10	9.60	1.50	8.90	1.80	4.80	0.80	5.10	0.80	
S		37.46	34.79	78.81	9.46	37.63	8.06	1.79	7.75	1.21	7.26	1.43	4.00	0.60	3.89	0.59	
SD		4.56	4.38	10.20	1.18	5.63	1.06	0.19	1.04	0.15	0.99	0.20	0.56	0.11	0.60	0.11	
V		12.16	12.58	12.94	12.48	14.95	13.14	10.55	13.43	12.02	13.67	13.91	13.89	17.82	15.52	19.17	
**PP**	**S**	**REY**	**LREY**	**MREY**	**HREY**	**LREY**	**MREY**	**HREY**	**C**	**U**	**E**	**C**		**U**	**E**	**REO**	**C_outl_**
		**[ppm]**				**[%]**			**[ppm]**			**[%]**				**[ppm]**	
Łaziska	Z1	220.84	163.94	47.50	9.40	74	22	4	80.90	57.00	82.94	37		26	38	261.00	0.98
	Z2	226.20	164.60	51.70	9.90	73	23	4	85.30	59.00	81.90	38		26	36	267.42	1.04
Siersza	Z3	222.65	157.75	55.10	9.80	71	25	4	85.20	58.00	79.45	38		26	36	263.61	1.07
	Z4	199.61	142.61	48.50	8.50	71	24	4	75.30	51.60	72.71	38		26	36	236.41	1.04
Jaworzno	Z5	220.82	156.32	54.00	10.50	71	24	5	86.40	54.60	79.82	39		25	36	261.38	1.08
	Z6	230.05	158.95	59.90	11.20	69	26	5	91.80	57.50	80.75	40		25	35	272.57	1.14
Łaziska	Z7	286.01	204.71	68.00	13.30	72	24	5	109.70	71.50	104.81	38		25	37	338.41	1.05
	Z8	271.59	201.09	59.10	11.40	74	22	4	100.20	71.30	100.09	37		26	37	320.98	1.00
Min.		199.61	142.61	47.50	8.50	69.09	21.51	4.20	75.30	51.60	72.71	36.63		24.73	35.10	236.41	0.98
Max		286.01	204.71	68.00	13.30	74.23	26.04	4.87	109.70	71.50	104.81	39.90		26.25	37.56	338.41	1.14
S		234.72	168.75	55.48	10.50	71.85	23.68	4.47	89.35	60.06	85.31	38.08		25.60	36.33	277.72	1.05
SD		28.91	22.15	6.74	1.47	1.74	1.55	0.25	11.01	7.37	11.08	1.09		0.59	0.74	34.10	0.05
V		12.31	13.13	12.16	14.03	2.43	6.54	5.68	12.33	12.27	12.99	2.86		2.32	2.05	12.28	4.74

PP, power plant; S, sample; C_outl_, outlook coefficient; REOs, rare earth elements oxides; C, critical; U, non-critical; E, excessive; Min., minimum; Max., maximum; S, average; SD, standard deviation; V, coefficient of variation.

## Data Availability

The original contributions presented in the study are included in the article, further inquiries can be directed to the corresponding author/s.
